# Supplementary material for: Analysis of Medical Services for Insomnia in Korea: A Retrospective, Cross-Sectional Study Using the Health Insurance Review and Assessment Claims Data
Source: Healthcare (Basel). 2021 Dec 22;10(1):7. doi: 10.3390/healthcare10010007 (PMC8775632; doi:10.3390/healthcare10010007)
Supplement: Supplementary file 1 [file healthcare-10-00007-s001.zip › healthcare-1462469-supplementary.pdf]

Supplementary Table S1. Characteristics of healthcare utilization.

Category		Total (2010-2016)		Western Medicine (2010-2016)		Korean Medicine (2010-2016)		P-value*
		No. of cases	%	No. of cases	%	No. of cases	%	
Type of visit	Outpatient	338,984	99.85	283,526	99.84	55,458	99.92	<.0001
	Inpatient	508	0.15	462	0.16	46	0.08	
Medical institution	Tertiary hospital/general hospital/hospital	43,753	12.89	43,608	15.36	145	0.26	<.0001
	Clinic	240,264	70.77	240,264	84.6	-	-	
	KM hospital	1,708	0.5	116	0.04	1,592	2.87	
	KM clinic	537,67	15.84	-	-	53,767	96.87	

KM=Korean Medicine; *Chi-square test;

Supplementary Table S2. Specific interventions for insomnia of Western and Korean medicine.

Type	Category	No. of services	Total expenses	No. of patients	Annual expenses per service	Annual expenses per patient
WM	Individual psychotherapy (supportive therapy)	63,478	$786,797.12	12,568	12.39	$62.60
	Individual psychotherapy (intensive therapy)	13,870	$323,320.61	5,288	23.31	$61.14
	Family therapy (individual treatment)	3,029	$44,123.78	974	14.57	$45.30
	Individual psychotherapy (intensive analytic therapy)	1,365	$49,853.81	797	36.52	$62.55
KM	Acupuncture	103,001	$408,779.61	10,029	3.97	$40.76
	Moxibustion	16,544	$44,772.76	3,542	2.71	$12.64
	Cupping	11,254	$50,319.11	2,587	4.47	$19.45
	Individual psychotherapy (Ijeong-byeongi therapy)	86	$1,008.39	31	11.73	$32.53
	Individual psychotherapy (Jieon-goron therapy)	43	$523.94	18	12.18	$29.11
	Individual psychotherapy (Gyeongja-pyeongji therapy)	42	$1,184.12	16	28.19	$74.01
	Individual psychotherapy (Oji-sangseung therapy)	31	$1,041.67	22	33.60	$47.35

WM=Western Medicine; KM=Korean Medicine; All cost-related results presented in this study were converted to the 2020 level based on the healthcare and medical service price index, adjusted for healthcare inflation rate and KRW:USD exchange rate (see Supplementary Table 4).

Supplementary Table S3. Anatomical Therapeutic Chemical Classification code by insomnia drug category.

Category	ATC codes
Hypnotics and sedatives	N05C
Antipsychotic drugs	N05A
Antianxiety drugs	N05B
Antidepressants	N06A
Antiseizure drugs	N03A, N04A
Antihistamines	R06A, R03D
Digestive and metabolic drugs	A02A, A02B, A02X, A03A, A03F, A04A, A16A
Musculoskeletal	M01A, M03B, N02A, N02B
Respiratory	R01B, R05C, R05D, R05F
Cardiovascular	C03A, C03C, C04A, C07A, C08C, C10A
Other	A10B, A11D, B01A, B05X, G03C, G04C, H02A, J01C, J01D, J01F, N01B, N06B, N06D, N07A, N07B, S01X

ATC=Anatomical Therapeutic Chemical Classification

Supplementary Table S4. Annual average KRW-USD exchange rate and price index of health expenses.

Year	KRW/USD	Healthcare & medical service price index
2010	1156.00	0.9164
2011	1107.99	0.9325
2012	1126.76	0.9410
2013	1095.04	0.9444
2014	1053.12	0.9510
2015	1131.52	0.9629
2016	1160.41	0.9725
:	:	:
**2020**	**1180.01**	**1.0000**

All cost-related results presented in this study were converted to the 2020 level based on healthcare and medical service price index adjusted for healthcare inflation rate and KRW:USD exchange rate; This information is available on the following site: Korean Statistical Information Service (http://kosis.kr);

Supplementary Figure S1. General medical service use for insomnia - Number of patients.

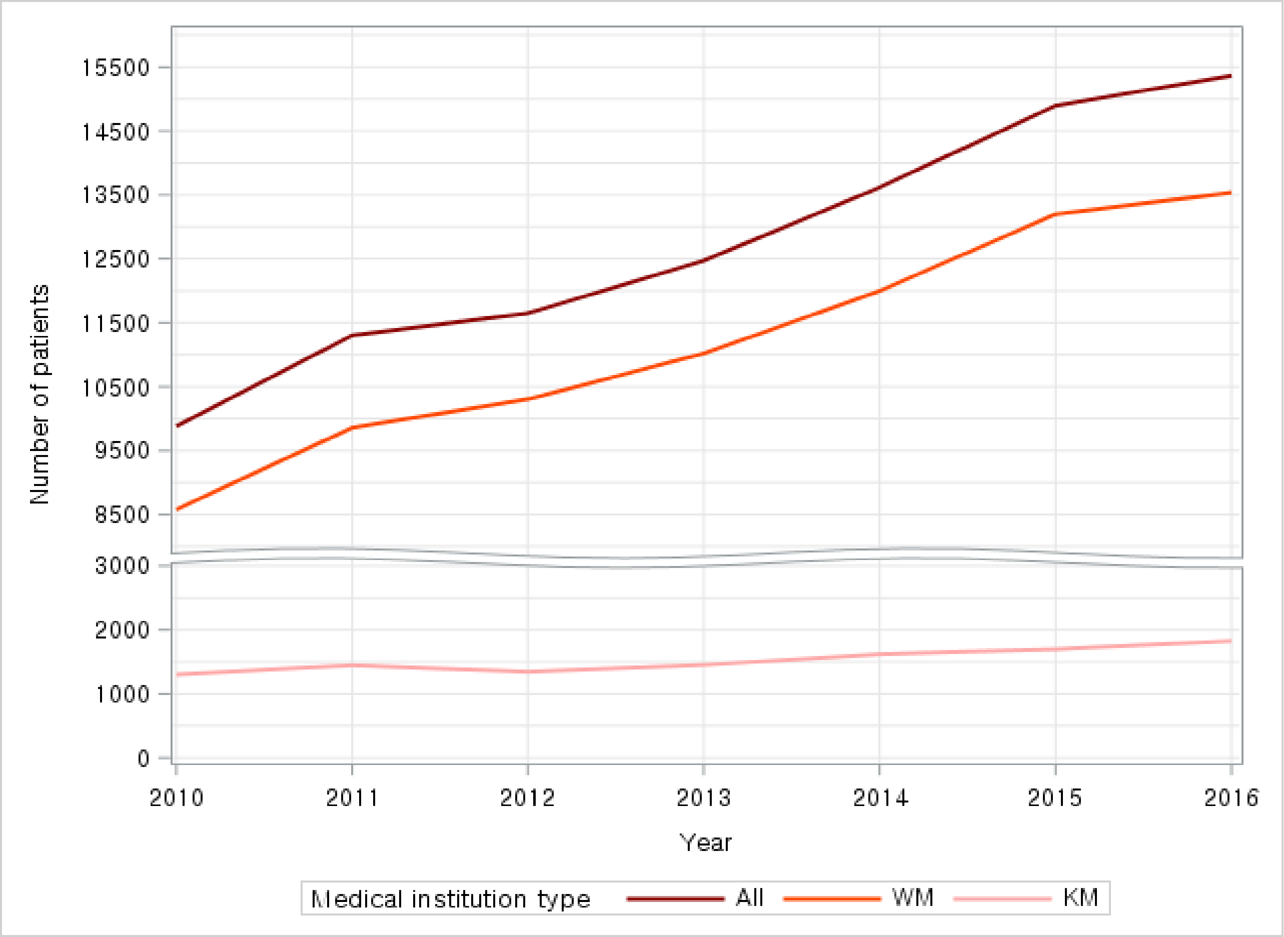

WM=Western Medicine; KM=Korean Medicine

Supplementary Figure S2. General medical service use for insomnia - Total expenses

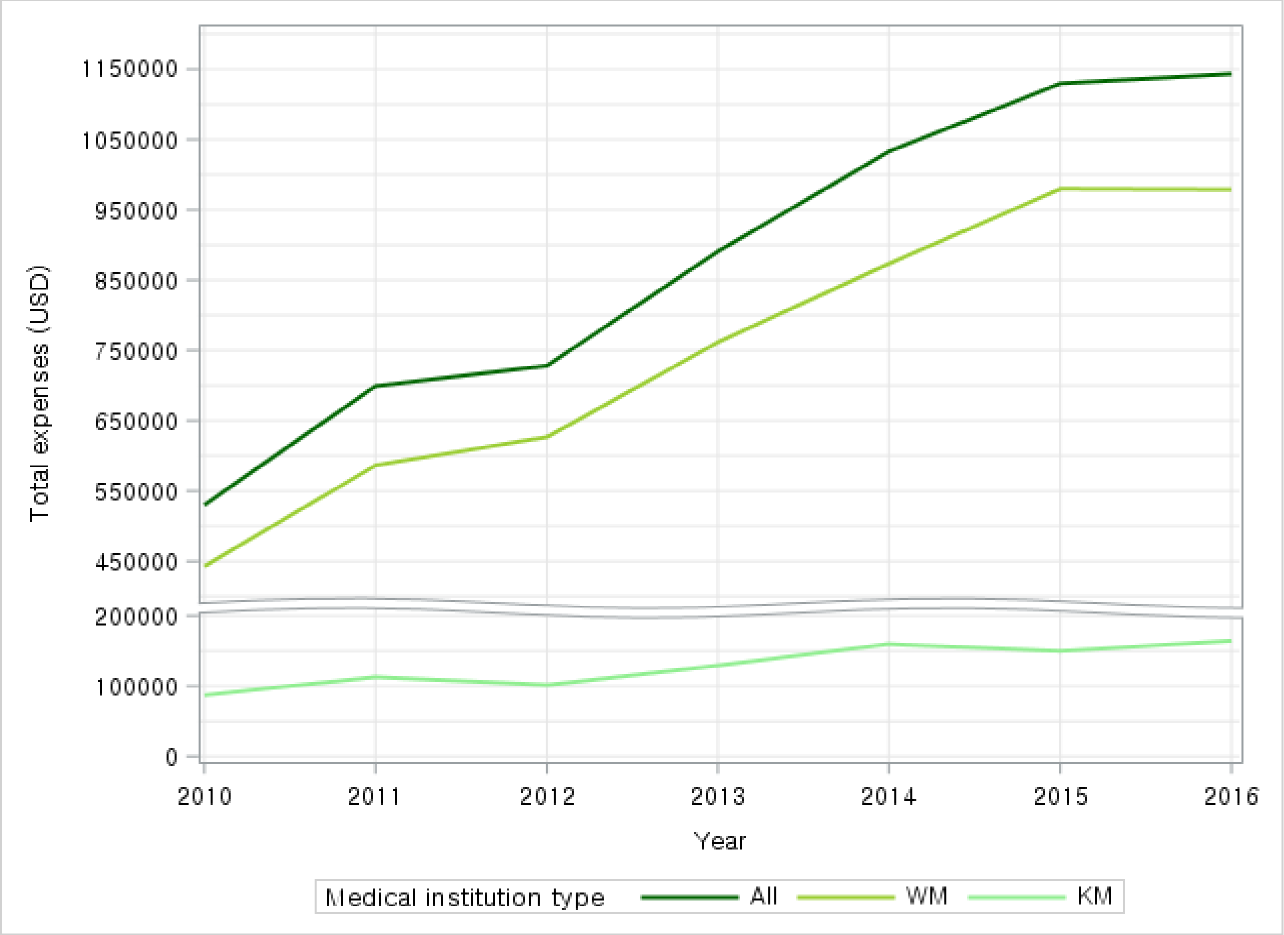

WM=Western Medicine; KM=Korean Medicine

Supplementary Figure S3. General medical service use for insomnia - Annual expenses per patient

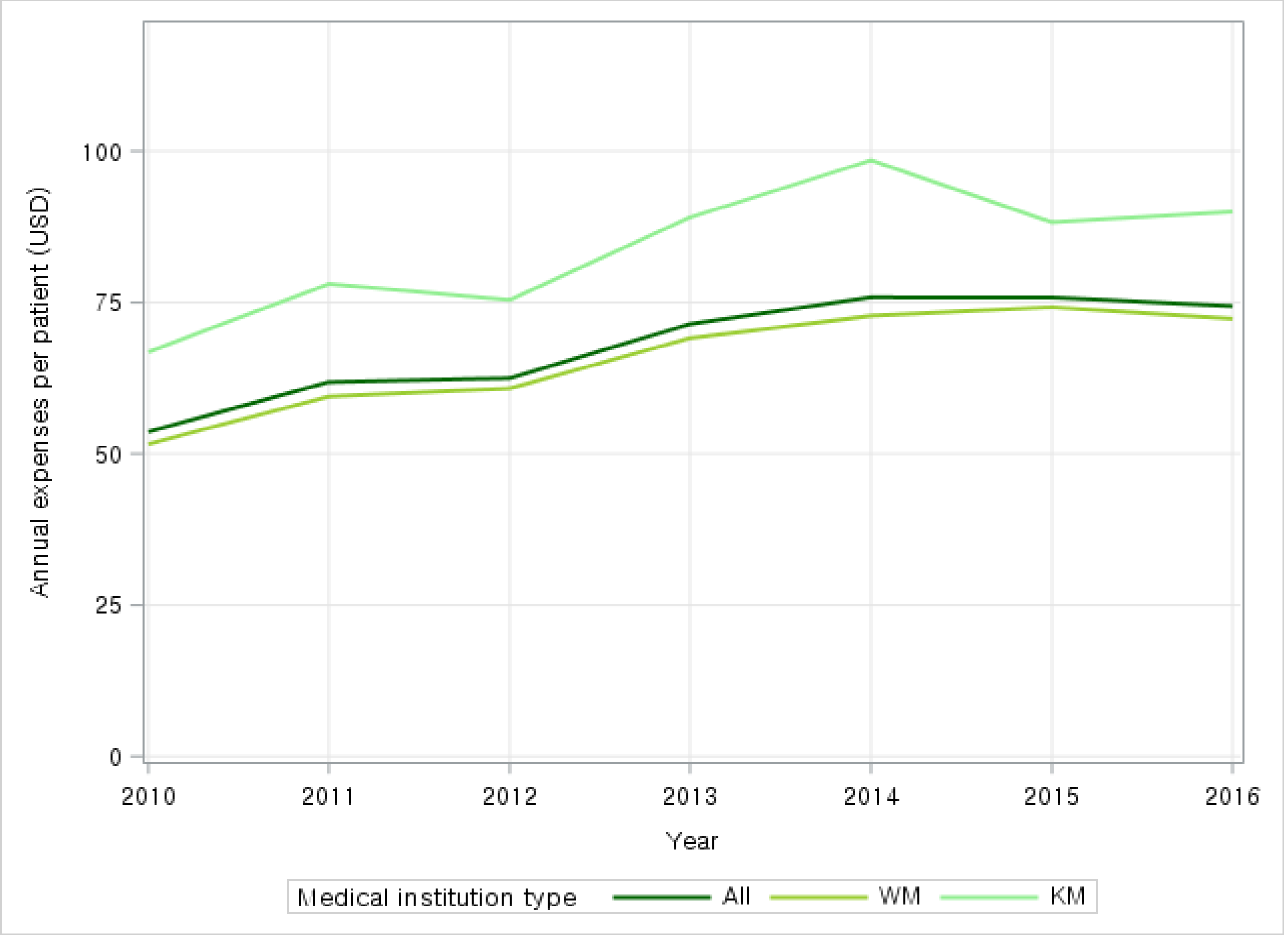

WM=Western Medicine; KM=Korean Medicine

Supplementary Figure S4. General medical service use for insomnia - Annual visits per patient

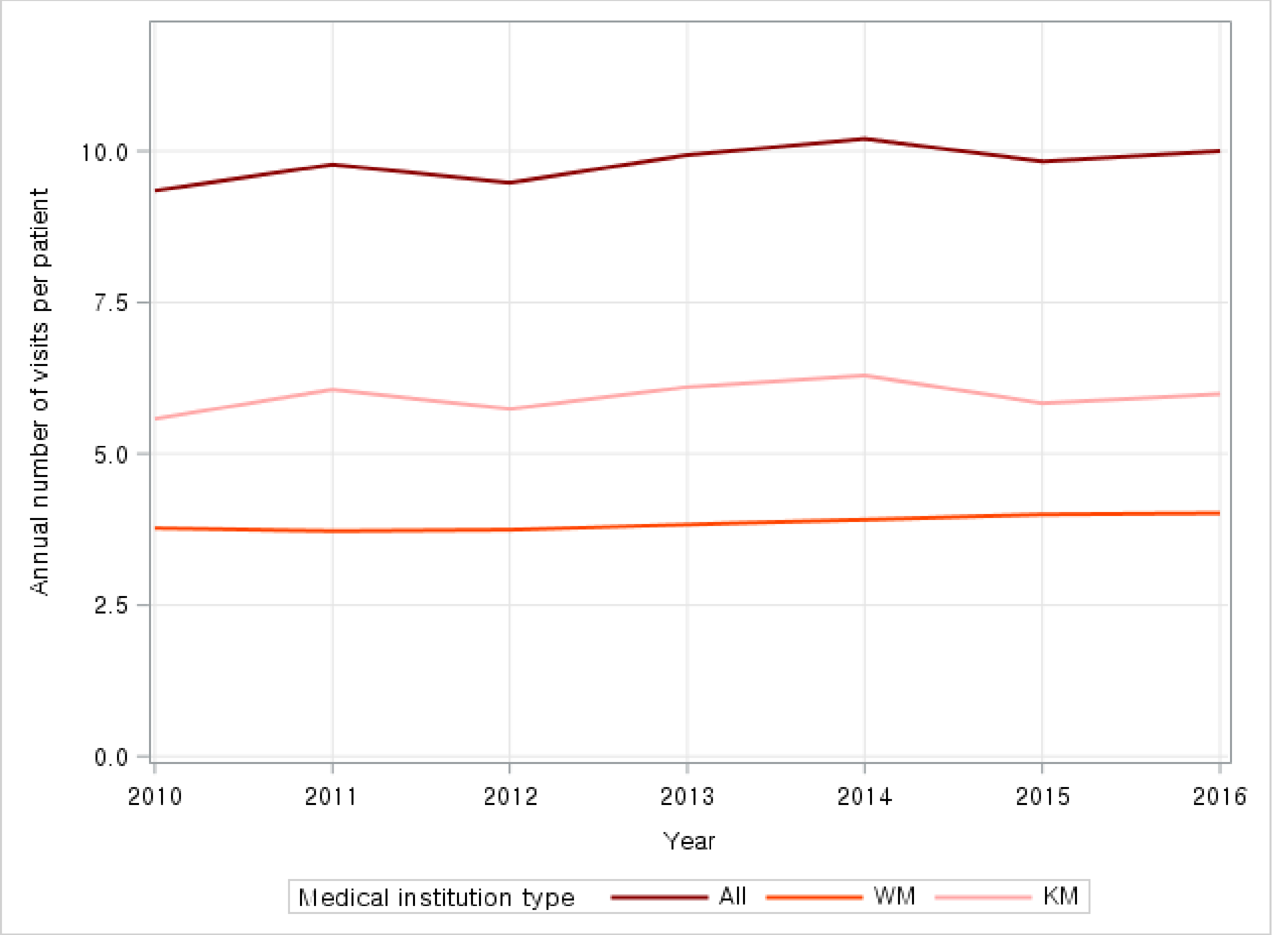

WM=Western Medicine; KM=Korean Medicine
